# Effects of cortisol administration on craving in heroin addicts

**DOI:** 10.1038/tp.2015.101

**Published:** 2015-07-28

**Authors:** M Walter, D Bentz, N Schicktanz, A Milnik, A Aerni, C Gerhards, K Schwegler, M Vogel, J Blum, O Schmid, B Roozendaal, U E Lang, S Borgwardt, D de Quervain

**Affiliations:** 1 Psychiatric University Clinics Basel, University of Basel, Basel, Switzerland; 2 Division of Cognitive Neuroscience, Department of Psychology, University of Basel, Basel, Switzerland; 3 Division of Molecular Neuroscience, Department of Psychology, University of Basel, Basel, Switzerland; 4 Department of Cognitive Neuroscience, Radboud University Medical Center, Donders Institute for Brain, Cognition and Behaviour, Radboud University Nijmegen, Nijmegen, Netherlands; 5 Transfaculty Research Platform, University of Basel, Basel, Switzerland

## Abstract

Heroin dependence is a severe and chronically relapsing substance use disorder with limited treatment options. Stress is known to increase craving and drug-taking behavior, but it is not known whether the stress hormone cortisol mediates these stress effects or whether cortisol may rather reduce craving, for example, by interfering with addiction memory. The aim of the present study was to determine the effects of cortisol administration on craving in heroin-dependent patients and to determine whether the effects depend on the daily dose of heroin consumption. We used a double-blind, placebo-controlled, cross-over study in 29 heroin-dependent patients in a stable heroin-assisted treatment setting. A single oral dose of 20 mg of cortisol or placebo was administered 105 min before the daily heroin administration. The primary outcome measure was cortisol-induced change in craving. Secondary measures included anxiety, anger and withdrawal symptoms. For the visual analog scale for craving, we found a significant interaction (*P*=0.0027) between study medication and heroin-dose group (that is, daily low, medium or high dose of heroin). Cortisol administration reduced craving in patients receiving a low dose of heroin (before heroin administration: *P*=0.0019; after heroin administration: *P*=0.0074), but not in patients receiving a medium or high dose of heroin. In a picture-rating task with drug-related pictures, cortisol administration did not affect the ratings for the picture-characteristic craving in all the three heroin-dose groups. Cortisol also did not significantly affect secondary outcome measures. In conclusion, a single administration of cortisol leads to reduced craving in low-dose heroin addicts. The present findings might have important clinical implications with regard to understanding stress effects and regarding treatment of addiction.

## Introduction

Opioid dependence, most commonly manifested as heroin dependence, is estimated to affect between 13 and 22 million persons worldwide.^1^ The risks of heroin dependence include fatal overdoses, infections (including endocarditis, human immunodeficiency virus infection and hepatitis C virus infection), social disintegration, violence and crime.^2^ Heroin dependence is generally known to be a chronically relapsing disorder that is characterized by compulsive drug use and loss of control over drug intake.^3^ The compulsion to use heroin is frequently driven by craving—a subjective experience of wanting to use and re-experiencing the positive effect of the drug.^4, 5^ Clinical research has demonstrated that opioid maintenance programs with regular opioid administration, including pharmaceutical heroin (diacetylmorphine), produce favorable treatment retention and reduce illicit opioid use in heroin-dependent patients.^6^ However, a substantial fraction of patients, especially during methadone maintenance treatment, continue to experience heroin craving and show illicit heroin use.^7^

Acute withdrawal in heroin addiction is accompanied by a negative affective state characterized by dysphoria, irritability, anxiety, as well as abnormal stress reactivity that drives drug seeking through negative reinforcement mechanisms.^8, 9^ For instance, abstinent heroin-dependent individuals show elevated stress reactivity, which is related to heightened craving and symptoms of withdrawal.^10^ Indeed, an activation of the hypothalamic–pituitary–adrenocortical axis and elevated glucocorticoid (that is, cortisol in humans) levels have been reported during opioid withdrawal syndromes,^11^ while opioid agonists were associated with a reduction in cortisol secretion.^12^ Furthermore, we found suppressed salivary cortisol concentrations,^13^ reduced craving scores and amygdala activity after opioid administration in heroin-dependent patients.^14, 15, 16^ Moreover, the opioid partial agonist buprenorphine has been found to dampen responses to psychosocial stress in healthy humans.^17^

Stress (that is, stressful live events and stressful conditions) has been found to increase craving and the risk of relapse to opioid use.^18, 19, 20^ It is not known, however, whether glucocorticoids are involved in mediating these stress effects. In case they are, the administration of glucocorticoids in heroin addicts could result in increased craving. On the other hand, exogenous glucocorticoid administration has been shown to induce temporary reduction in the retrieval of different forms of memory, including aversive memory in anxiety disorders,^21^ and could therefore also reduce addiction-related memory. The neurobiology of drug addiction shares striking commonalities with the neurobiology of learning and memory, including shared neural circuitries and molecular mechanisms.^22, 23^ In line with these neurobiological models of addiction memory, studies in addicted patients have indicated that the evocation of conditioned responses by drug-associated stimuli is important in the maintenance of drug use and relapse.^24, 25^ Thus, memory has an important role in addiction as it stores the powerful incentives associated with drug taking that produce a strong feeling of craving.^5, 22, 24, 26^ Therefore, the administration of cortisol could result in reduced retrieval of addiction memory and, thereby, reduce feelings of craving.

To date, it is unknown whether exogenous glucocorticoids would increase or rather decrease craving in heroin-dependent patients. Because of the considerable clinical implications with regard to understanding stress effects and regarding treatment of addiction, the present study examined the effects of a single administration of cortisol on craving in heroin-dependent patients. We also examined whether cortisol effects depend on the daily dose of heroin consumption as glucocorticoids have been shown to interact with the opioid system.^27^

## Materials and methods

### Participants

All the participants were recruited from a standardized heroin-assisted treatment protocol conducted at the treatment center JANUS, Psychiatric University Clinics Basel, Basel, Switzerland. The heroin-assisted treatment consists of the prescription of pharmaceutical heroin (diacetylmorphine) twice a day combined with additional medical and psychosocial services.^28^ Additional services include medical care for somatic and psychiatric conditions, weekly individual and group support sessions and help from social workers.

Thirty-one participants with opioid dependence according to ICD-10 gave written informed consent to take part in the study. Sample size was estimated on the basis of the assumption of a medium effect size at an alpha level of 0.05 and a power of 0.8. Two participants had to be excluded after study enrollment, one participant because of an additional psychiatric axis I disorder identified during the medical examination for study participation and the other participant because of illicit co-consumption of a not-prescribed tranquilizer (flunitrazepam) during the first study day. The remaining 29 participants (7 females, 22 males) completed the study and were entered in the analyses (see Supplementary Information for flow diagram Supplementary Figure S1). Mean age was 42.4 (s.d. 7.9) years with a mean duration of heroin consumption of 23.3 (s.d. 9.0) years and a current daily intake of 395.8 (s.d. 171.1) mg heroin. Two out of seven females took oral contraceptives (for additional demographic and psychometric information, see Table 1).

Inclusion criteria consisted of an age older than 18 years, meeting criteria for opioid dependence according to ICD-10 and having a past history of intravenous heroin consumption with an unchanged heroin intravenous substitution for at least 3 months. In addition, participants had to be able to control parallel consumption of street drugs and were told to abstain from illicit drug consumption for the duration of study participation. Blood alcohol level had to be below 30 ml on both the study days. A urine drug test (6-Panel Drug Test for screening for amphetamine, benzodiazepine, cocaine/benzoylecgonine, methadone, opiates/morphine, cannabis; Stephany Diagnostika, Grenzach-Wyhlen, Germany) and a breathalyzer test (Dräger Alcotest 6510 Fuel Cell Breathalyzer, Lübeck, Germany) were performed at the beginning of both the study days. Drug screen results were considered as additional covariates in the statistical model (see Results).

Exclusion criteria were a recent history of systemic or topical glucocorticoid therapy or hypersensitivity to glucocorticoids, an axis I disorder besides opioid dependence, current medical condition (such as infectious disease), inability to read and understand the study consent form, pregnancy or lactating. The local ethic committee and the Swiss agency for the authorization and supervision of therapeutic products (Swissmedic, Bern, Switzerland) approved the study. The study was registered with ClinicalTrials.gov (http://clinicaltrials.gov/ct2/show/NCT01718964?term=de+quervain&rank=5). The blinding was maintained throughout the study. All the participants received vouchers for local supermarkets (value 80 Swiss francs) as compensation for their participation.

### Procedure and measurements

**Figure 1:**
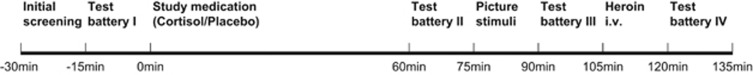
Course of the study. The x axis illustrates the time line. Study medication (that is, cortisol or placebo) is administered at 0 min.

The study took place on two study days (120–135 min duration each) between 1230 and 1630 h at the JANUS center of the Psychiatric University Clinics Basel, Basel, Switzerland in November 2012–July 2013. Both study days were at least 1 week, but no longer than 3 weeks, apart. Besides the initial screening at the beginning of the first study day, both study days had the same procedure (see Figure 1). First, baseline saliva was sampled and psychometric measures with the study test battery were assessed. Afterwards, the study medication was administered followed by a 1-h resting period allowing the absorption of the study medication. The study test battery consisted of self-report measures, saliva samples and examination of vital signs. This test battery was given a total of four times, I–IV, on both study days (I: 15 min before administration of study medication, II: 60 min after administration of study medication, III: 90 min after administration of study medication, IV: 120 min after administration of study medication directly after heroin administration). Furthermore, 75 min after the administration of study medication, a picture-rating task was administered. The goal of this task was to investigate whether cortisol affects craving ratings of drug- and nondrug-related pictures (see below).

#### Study medication

The participants were allocated randomly by time of study entry to receive either oral cortisol (20 mg, two tablets each of 10 mg of hydrocortisone; Galepharm, Küsnacht, Switzerland) or placebo (two similar looking tablets) at the first study day. This dose of cortisol has been used in previous studies investigating the effects of a single administration of cortisol on phobic fear.^29^ After a washout period of 7–21 days, participants received on the second study day the treatment (cortisol or placebo) that they had not received on the first study day. University Hospital Pharmacy Basel prepared and blinded study medication (double-blind, placebo-controlled crossover design).

#### Saliva measurements

Saliva was collected with Salivette (Sarstedt, Rommelsdorf, Germany). Saliva samples were taken 15 min before (−15) and 60 min after (+60) administration of study medication, directly after stimuli presentation (+90) and after heroin intravenous (+120). Salivary cortisol was analyzed as described before.^29^

#### Self-report measures of the test battery

The test battery was administered at four time points on both the study days and included a primary outcome measure (that is, craving) and secondary outcome measures (that is, state anxiety, state anger and withdrawal symptoms). Craving: craving was the primary outcome measure and assessed by visual analog scales for craving (VASC). Visual analog scales have been successfully used to measure the changes in emotional states.^30^ Participants had to indicate the intensity of their craving on a continuous horizontal line (10 cm) with the two end points—not at all (0) and very strong (10). Craving was also measured with the ‘Desire to use heroin' scale of the Heroin Craving Questionnaire (HCQ). The scale originally consists of nine items scored between 1 and 7 (total score ranges between 9 and 63, higher scores indicate stronger craving).^5^ To avoid confusion, we excluded questions that contained double negations, resulting in only five items (item 11, 17, 23, 39, 42) with a total score between 5 and 35. State anxiety: state anxiety was measured with the German version of the State–Trait Anxiety Inventory. The state scale of STAI consists of 20 items scored between 1 and 4 (total score ranges between 20 and 80, higher scores indicate higher state anxiety). State anger: state anger was measured with the Anger Expression Inventory (STAXI). The state scale of STAXI consists of 10 items scored between 1 and 4 (total score ranges between 10 and 40, higher scores indicate higher state anger). Withdrawal symptoms: withdrawal symptoms were measured by nine selected items of the Short Opiate Withdrawal Scale (SOWS) scored between 0 and 3 (total score ranges between 0 and 27, higher scores indicate more and/or stronger symptoms).

#### Picture-rating task

The goal of this task was to investigate whether cortisol affects craving ratings of drug- and nondrug-related pictures. Picture stimuli were presented in a darkened room on one laptop in groups of maximal two participants at the same time. The picture stimuli consisted of drug-related pictures and three categories of nondrug-related pictures (that is, neutral, negative and positive photographs), with four different pictures per category. Two comparable versions were used for the two study days. The pictures were pseudo-randomized according to the rule that pictures of the same category never followed one another. Pictures were presented in the same order for all the participants. Each picture was presented for 4 s and followed by a prompt to rate it for the picture-characteristic craving by means of a visual analog scale. All the pictures except the drug-related pictures were taken from the International Affective Picture System. The drug-related pictures were nonstandardized pictures showing themes related to drug use, like drug injection, preparation of drugs and typical drug-consumption situations.

### Statistical analysis

The five variables (VASC, HCQ, STAI, STAXI, SOWS) were measured at four different time points: baseline (I), 60 min (II) and 90 min (III) after study medication intake and after heroin consumption (IV) as described above (see also Figure 1). For each of the five variables of interest, we tested the correlation structure for the four repeated measurements and observed a decrease of correlation after heroin consumption. Therefore, we calculated two separate models (1) for the two time points before heroin consumption (II, III) and (2) after heroin consumption (IV). Analyses were done in R (http://www.r-project.org/). We applied linear models and linear mixed models (nlme-package) in combination with analysis of variance (SS II), when necessary. In case of repeated measurements, participant was included as the random effect of the mixed model. Dependent variables were the measurements of the five variables of interest. Independent variable was the study medication (placebo or cortisol).

To investigate whether cortisol effects depend on the daily heroin dose, we divided the sample into three nearly equally sized heroin groups. This group assignment allowed us to test not only for linear, but also for nonlinear relationships between heroin consumption, study medication and our variables of interest. Because the administered intravenous heroin and the administered oral heroin medicament do not contain exactly the same amount of active pharmaceutical ingredient, we quantified the current daily heroin consumption according to the formula: daily heroin consumption=(daily intravenous heroin medication × 174.2)/200+(daily oral heroin medication × 182)/200. The three groups were as follows: participants with low-dose (range 113–305 mg, mean 235 mg, *N*=10), medium-dose (range 330–451 mg, mean 379 mg, *N*=9) and high-dose (range 478–964 mg, mean 572 mg, *N*=10) heroin consumption. This allocation resulted in the maximal separation between the groups with regard to the daily heroin consumption values. Group assignment was entered as a factor in the model. We tested for main effects of study medication and for interaction effects between heroin group and study medication on our dependent variables. The baseline values (I) of the variables of interest were included as covariate in all the models. We also included treatment order as covariate in all the models. In case of repeated measurement, the time point of measurement (60 min or 90 min) was included as covariate. Due to the five variables of interest and two time points tested, we set the significance threshold to *P*<0.005 (Bonferroni correction for 10 independent tests). In case of a Bonferroni-corrected significant interaction between heroin group and study medication, we performed *post hoc* tests for each heroin group separately (nominal significance threshold *P*<0.05).

In addition, we analyzed craving ratings of drug-related and nondrug-related pictures. This craving value was the dependent variable of the linear model. Again, we tested for the independent main effect of medication and the interaction between medication and heroin group. Treatment order was included as a covariate.

## Results

### Effects of study medication on salivary cortisol levels

There was a significant main effect of study medication (cortisol/placebo) on salivary cortisol levels, with significant higher cortisol levels under cortisol treatment at the three time points (that is, at 60 min, 90 min and 120 min) after medication (F_(1,138)_=960.09, *P*<0.00001; Supplementary Figure S2). In addition, we detected a significant main effect of the three heroin groups on cortisol levels (F_(2,25)_=3.69, *P*=0.039). *Post hoc t*-tests showed significantly higher cortisol levels in the medium-dose heroin group compared with the high-dose heroin group (F_(1,16)_=8.00, *P*=0.012), but no significant different cortisol levels between the low- and medium-dose heroin groups (*P*=0.37) or between the low- and high-dose heroin groups (*P*=0.12). However, we did not find a significant interaction effect between heroin-dose group and study medication on cortisol levels (F_(2,134)_=1.56, *P*=0.21). Furthermore, there were no differences between the heroin groups with regard to cortisol levels at baseline or cortisol levels in the course of the experiment in the placebo condition (*P*⩾0.12).

### Effects of study medication on primary and secondary outcome measures

**Figure 2:**
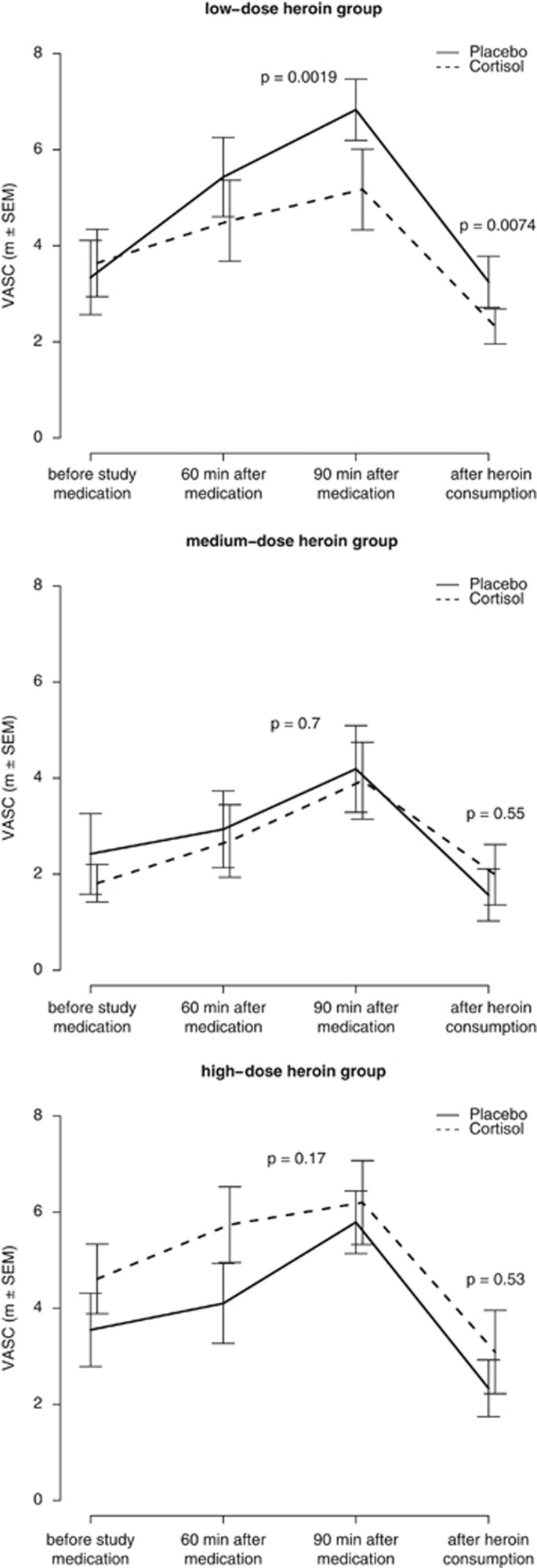
Effects of cortisol administration on craving. Means and standard errors are displayed. Study medication was cortisol/placebo. The solid line represents the placebo medication, whereas the dotted line represents the cortisol medication. VASC, visual analog scale for craving.

For the primary outcome craving, as quantified by VASC, there was a significant interaction (F_(2,82)_=8.00, *P*=0.00067) between medication and heroin group (that is, low-, medium-, high-dose) before heroin consumption, which survived Bonferroni correction. We applied *post hoc* tests for the three heroin groups to test for an influence of medication on VASC before heroin consumption (that is, 60 min and 90 min after the administration of study medication). Cortisol administration reduced VASC in the low-dose heroin group (*t*_(27)_=3.45, *P*=0.0019; see Figure 2), but not for the medium-dose (*t*_(24)_=0.4, *P*=0.70) or high-dose heroin group (*t*_(27)_=−1.42, *P*=0.17). For the interaction between medication and heroin group at the time point after heroin consumption, there was only a trend (*F*_(2,24)_=2.71, *P*=0.087). For the sake of completeness, we ran the *post hoc* tests also for the time point after heroin consumption, which showed the same pattern, meaning that cortisol administration reduced craving in the low-dose heroin group (*t*_(8)_=3.56, *P*=0.0074, see Figure 2), but not in the medium-dose (*t*_(7)_=−0.63, *P*=0.55) or high-dose heroin group (*t*_(7)_−0.66, *P*=0.53).

In an additional analysis, we included all three time points at once in the same linear mixed model (that is, 60 and 90 min after medication and after heroin consumption). Here again, we identified a significant interaction between medication and heroin group on VASC (F_(2,138)_=6.19, *P*=0.0027).

We also tested for an exclusive linear relationship regarding the interaction between medication and heroin consumption. Therefore, we entered the amount of current daily heroin consumption, instead of heroin group, in the model. We still observed a significant interaction between medication and heroin dose for VASC (F_(1,83)_=4.59, *P*=0.035). To analyze whether the three groups differed in the amount of drug co-consumption, we calculated *χ*^2^ tests for each screened co-used drug. We found a significant association between heroin group and benzodiazepine co-consumption (*χ*^2^(2)=12.50, *P*=0.0019), as well as a significant association between heroin group and methadone co-consumption (*χ*^2^(2)=10.29, *P*=0.0058). No significant association was found between cocaine co-consumption and heroin group as well as cannabis co-consumption and heroin group (both *P*>0.46; for a descriptive overview, refer to Supplementary Table S3). Furthermore, the three groups did not differ in the amount of smoked cigarettes during the resting period (F_(2,26)_=0.78, *P*=0.47). In addition, we investigated whether the interaction between the three heroin groups and medication on VASC might have been owing to confounding variables, such as employment status, duration of heroin dependency, benzodiazepine co-consumption, cocaine co-consumption, cannabis co-consumption, amount of smoked cigarettes during the resting period, additional treatment with methadone, age or sex. The interaction between heroin group and study medication was still observed when entering these covariates separately in the model (medication × heroin group *P*<0.01 for all the analyses). The interaction between heroin group and study medication on HCQ did reach nominal significance (*P*=0.012), but did not survive Bonferroni correction for multiple comparisons. There were no significant main effects of study medication on the five variables of interest before and after heroin consumption (see Table 2). Furthermore, for none of the secondary measures (STAI, STAXI, SOWS), we found significant main effects of study medication or study medication × heroin group interaction effects, which survived Bonferroni correction (see Table 2).

Moreover, we analyzed whether the duration of opiate abstinence interfered with cortisol-related effects on craving. The mean abstinence duration between the last opiate administration in the morning and the beginning of the baseline measures was 5.95 h (s.d.=0.79). There was no significant main effect of heroin group on abstinence duration (F_(2,26)_=1.96, *P*=0.16) and no significant interaction between medication and heroin group on abstinence duration (F_(2,84)_=0.90, *P*=0.41). Moreover, the significant interaction between heroin group and medication on VASC was still observed when entering the covariate abstinence duration in the model (F_(2,80)_=7.40, *P*=0.0011).

To rule out that limited cortisol uptake has masked potential effects in the high-heroin group, we entered the salivary cortisol levels, instead of the factor medication, in the model. We detected a significant interaction between cortisol levels and heroin groups on VASC at the time point before heroin consumption (F_(2,82)_=8.94, *P*=0.0003), which survived Bonferroni correction. *Post hoc* tests for each heroin group separately revealed for the low-dose group a significant main effect of cortisol level on VASC (*t*_(27)_=−3.97, *P*=0.0005), with cortisol reducing craving as quantified by VASC, but no effect of cortisol level on VASC for the medium-dose (*t*_(23)_=0.13, *P*=0.90) or high-dose heroin group (*t*_(23)_=1.05, *P*=0.31) was detected. There was no Bonferroni-corrected significant interaction between cortisol level and HCQ (F_(2,81)_=4.30, *P*=0.017), STAI (F_(2,81)_=4.78, *P*=0.01), STAXI (F_(2,81)_=4.16, *P*=0.019) or SOWS (*P*=0.38) at the time point before heroin consumption, and no significant main effect of cortisol level on any of these variables (*P*>0.60). For the time point after heroin consumption, no Bonferroni-corrected significant interaction between cortisol and heroin group (all *P*>0.27) or main effects of cortisol (all *P*>0.019) were detected.

We initially divided the sample into three heroin groups, which allowed us to test not only for linear, but also for nonlinear relationships between heroin consumption, study medication and our variables of interest. We additionally divided our sample into two equally sized groups, by means of a median split, to achieve more power. The lower-dose heroin group (range 113–370 mg, mean 268 mg, *N*=14) and the higher-dose group (range 382–964 mg, mean 515 mg, *N*=15). There was a significant interaction between medication and heroin group (that is, lower dose and higher dose) on VASC (F_(1,83)_=24.37, *P*=0.0000008), on HCQ (F_(1,83)_=14.38, *P*=0.0003) and on STAI (F_(1,82)_=13.43, *P*=0.0004) before heroin consumption, which survived Bonferroni correction. No significant Bonferroni-corrected interaction between medication and heroin group on SOWS or STAXI (all *P*>0.03) or main effects (all *P*>0.70) were observed. We applied *post hoc* tests for the two heroin groups separately to test for an influence of medication on VASC, HCQ and STAI before heroin consumption (that is, 60 min and 90 min after the administration of study medication). Cortisol administration significantly reduced craving as quantified by VASC (*t*_(38)_=3.92, *P*=0.0004) and by HCQ (*t*_(38)_=2.58, *P*=0.01), and reduced state anxiety (*t*_(38)_=2.39, *P*=0.02) in the lower-dose heroin group. There was a nominally significant main effect for enhanced VASC in the higher-dose group (*t*_(36)_=−2.48, *P*=0.02), HCQ (*t*_(36)_=−2.75, *P*=0.009) and STAI (*t*_(36)_=−2.48, *P*=0.01), indicating more craving and state anxiety due to cortisol administration for the higher-dose group. For the interaction between medication and heroin group on VASC at the time point after heroin consumption, there was only a trend (*F*_(1,25)_=4.61, *P_uncorrected_*=0.04) but not for HCQ, STAI, STAXI or SOWS (all *P*>0.21, main effects all *P*>0.1).

### Effects of study medication on the picture-rating task

In addition, we investigated the effects of the study medication on ratings of drug- or nondrug-related pictures with regard to the picture-characteristic craving. We did not find significant interactions between medication, picture type and heroin-dose group, between medication and picture type, or between medication and the three heroin-dose groups on VASC (*P*⩾0.27). Furthermore, there was no significant main effect of medication on VASC (F_(1,85)_=0.08, *P*=0.78), but a significant main effect of picture type, with higher craving ratings of drug-related pictures as compared with nondrug-related pictures (F_(1,85)_=62.71, *P*<0.001, see Supplementary Tables S1 and S2).

### Effects of study medication on adverse events

The rate of reported adverse events, which mainly consisted of headache, tiredness or agitation, did not differ significantly between the cortisol and placebo condition (6 out of 30 patients reported adverse events after placebo administration, while 8 out of 29 patients reported adverse events after cortisol administration; Chi-square test: *χ*^2^ (1)=1.04; *P*=0.31).

## Discussion

The present study revealed that a single oral administration of cortisol can significantly reduce craving in heroin-maintained patients. This craving-reducing effect of cortisol was heroin dose-dependent and only found in patients receiving low-dose daily heroin (that is, up to 305 mg per day). State anxiety, state anger and symptoms of opiate withdrawal were not significantly affected by cortisol.

Stress has been found to increase craving and the risk of relapse to opioid dependence.^18, 19^ Although animal studies have suggested that glucocorticoids might be involved in mediating the enhancing effects of stress on craving and relapse,^18, 31^ studies in humans failed to confirm this postulated role of glucocorticoids.^32, 33^ The present finding of a cortisol-induced reduction in craving suggests that glucocorticoids are not mediating the enhancing effect of stress on heroin craving, but rather act as a stress buffer. A possible mechanism for the craving-reducing effect of glucocorticoids may be their effects on memory retrieval. Glucocorticoids have been shown to reduce retrieval of previously acquired information in rodents and healthy humans^34, 35^ and there is evidence suggesting that emotionally arousing information is particularly sensitive to these glucocorticoid effects.^36^ Furthermore, there is evidence that glucocorticoids can also reduce the retrieval of aversive memory and enhance fear extinction in posttraumatic stress disorder and phobia.^29, 30, 37^

There is growing evidence that memory and addiction partly share neural circuitries and molecular mechanisms.^22^ It has been proposed that critical neuroadaptations for addiction render brain-reward systems hypersensitive to drugs and drug-associated stimuli.^26^ Importantly, the powerful incentives associated with drug taking that produce a strong feeling of craving^5^ are stored in memory; also referred to as addiction memory.^24^ Moreover, craving is triggered by contextual cues stored in memory.^38^ Therefore, in the present study, glucocorticoids may have reduced craving by interfering with the retrieval of addiction memory and/or contextual memory. According to this idea, cortisol should have also reduced the rating of drug-related pictures with regard to the picture-characteristic ‘craving'. However, we did not observe a cortisol effect on the appraisal of drug-related cues when they are explicitly presented. A possible explanation for the lack of cortisol effect in this situation might be that explicitly shown drug-related stimuli are too strong to be influenced by cortisol. Interestingly, a similar discrepancy has been found with declarative memory, where cortisol reduces memory retrieval in a free-recall task, but not in a recognition task that involves the explicit presentation of the stimuli.^35^

Alternatively, or in addition to the glucocorticoid effects on memory, cortisol may have exerted direct effects on the reward system, as previous studies have found that glucocorticoids can effect dopaminergic transmission and reward behavior.^39, 40^ However, the studies are not univocal with regard to the direction of effect. For example, a study in rats has shown that acute glucocorticoid administration acts in the nucleus accumbens to enhance dopamine signaling and potentiate reinstatement of cocaine seeking.^39^ In contrast, a study in healthy humans has shown that acute glucocorticoid administration induces a global downregulation of the brain's reward circuitry.^40^

Interestingly, we found the craving-reducing effect of cortisol only in patients with low-dose heroin consumption (113–305 mg per day). The three subgroups did not differ in age, gender distribution or duration of dependency. Also the salivary cortisol data at baseline or at the other time points of the experiment did not explain why only the low-dose group responded to cortisol administration. However, the medium- and high-dose heroin consumption groups had more frequently an unemployment status as compared with the low-dose consumption group, indicating a more severe substance use disorder, which is generally less responsive to regular treatment interventions.^41, 42^ Alternatively, higher doses may have interfered directly or indirectly with cortisol effects. For example, studies in rodents have shown that glucocorticoids interact with the opioid system to influence memory-retrieval mechanisms.^27^ Therefore, daily consumption of higher heroin doses might have disturbed this interaction through individual differences in sensitivity of the opioid system or drug-induced downregulation of opioid receptors. Furthermore, since nicotine is a strong modulator of the hypothalamic–pituitary–adrenocortical axis,^43^ group differences in the amount of daily cigarette smoking or in the smoking abstinence duration before testing might have influenced cortisol effects on craving. Unfortunately, these smoking data were not available, which represents a limitation of the present study. Finally, it is possible that higher doses of cortisol might have been needed to reduce craving in patients receiving higher heroin doses.

To conclude, we believe this is the first study to examine the acute effects of cortisol administration in a population of heroin-dependent patients in a controlled study design. It shows that a single administration of cortisol leads to reduced craving. Future studies will need to identify potential factors that influence cortisol effects on craving, such as sex-steroid hormones^44^ and to explore the mechanism and the therapeutic potential of glucocorticoids in drug addiction. In particular, it will be of considerable clinical interest to investigate the effects of repeated administration of glucocorticoids and whether glucocorticoids might enhance exposure-based therapy, as it has been shown in phobia.^29^ Moreover, it will be of interest to investigate whether cortisol might be suited to prevent relapse in abstinent patients.

## Figures and Tables

**Table 1:** Demographic and baseline variable of interest characteristics

	*All (*N=*29)*	*Low-dose heroin group (*N=*10)*	*Medium-dose heroin group (*N=*9)*	*High-dose heroin group (*N=*10)*	P*-values*
Females/males	7/22	3/7	3/6	1/9	0.56
Oral contraceptives yes/no	2/5	1/2	0/3	1/0	
Age	42.4 (7.9)	39.3 (8.6)	42.4 (7.5)	45.4 (7.1)	0.24
Heroin dose^a^	395.8 (171.1)	235.1 (70.2)	378.9 (35.3)	571.7 (147.1)	<0.000001
Additional methadone yes/no	6/23	3/7	1/8	2/8	0.85
Dependency duration	23.3 (9.0)	19.6 (8.7)	24.8 (7.6)	25.7 (10.0)	0.27
Abstinence duration	5.95 (0.79)	6.07 (0.79)	6.20 (0.50)	5.59 (0.89)	0.16
Employed/unemployed	11/18	7/3	3/6	1/9	0.02
BMI	26.1 (4.4)	26.1 (5.9)	25.6 (3.5)	26.5 (3.7)	0.92
VASC	3.3 (2.4)	3.5 (2.4)	2.1 (2.0)	4.1 (2.4)	0.12
HCQ	12.9 (7.1)	12.7 (7.1)	11.3 (6.7)	14.7 (7.4)	0.54
STAI	36.5 (11.1)	37.4 (9.8)	33.2 (10.6)	38.5 (12.7)	0.54
STAXI	11.5 (3.0)	11.1 (2.1)	10.7 (1.7)	12.7 (4.2)	0.2
SOWS	11.4 (3.8)	10.9 (1.9)	10.0 (2.2)	13.3 (5.5)	0.1

Abbreviations: BMI, body mass index; HCQ, heroin craving index; SOWS, opiate withdrawal index; STAI, state anxiety index; STAXI, state anger expression index; VASC, visual analog scale for craving.

a Current daily heroin dose in mg=(daily intravenous heroin medication × 174.2)/200+(daily oral heroin medication × 182)/200.

Demographic variables are the number of males and females; age in years; heroin dose indicates the current daily heroin dose in mg; additional methadone indicates the additional treatment with methadone; dependency duration indicates the duration of heroin dependency in years; abstinence duration indicates the mean abstinence duration in hours between the last opiate administration in the morning and the beginning of the baseline measures. Means of the baseline variable of interest values were calculated over the two testing days. Data presented as mean (s.d.). *P* indicates *P*-values of heroin group effect on baseline variables of interest values.

**Table 2:** Cortisol effects on clinical symptoms

*Variable*	P*-values*			
	*Time point 60 and 90 min after study medication* *(before heroin consumption)*		*Time point 120 min after study medication* *(after heroin consumption)*	
	*ME medication*	*Medication* × *heroin group*	*ME medication*	*Medication* × *heroin group*
VASC	0.32	0.00067*	0.8	0.087
HCQ	0.98	0.012	0.11	0.46
STAI	0.75	0.033	0.34	0.39
STAXI	0.67	0.025	0.095	0.62
SOWS	0.94	0.25	0.31	0.81

Abbreviations: HCQ, heroin craving index; ME, main effect; SOWS, opiate withdrawal index; STAI, state anxiety index; STAXI, state anger expression index; VASC, visual analog scale for craving.

*P<0.005, Bonferroni-corrected α level.

For craving (VASC), there was a significant interaction between study medication and heroin group (low, medium, high dose) before heroin consumption, which survived Bonferroni correction for 10 comparisons (*P*<0.005).
