# Supplementary material for: Effects of cortisol administration on craving in heroin addicts
Source: Transl Psychiatry. 2015 Jul 28;5(7):e610–. doi: 10.1038/tp.2015.101 (PMC5068724; doi:10.1038/tp.2015.101)
Supplement: Supplementary Tables [file tp2015101x1.doc]

| Table S1. No cortisol effects on craving related to viewing drug-related and non-drug-related pictures | |
| --- | --- |
| Effects on VASC | p |
| Medication X heroin group X picture type | 0.75 |
| Medication X heroin group | 0.27 |
| Medication X picture type | 0.57 |
| Heroin group X picture type | 0.49 |
| ME medication | 0.78 |
| ME heroin group | 0.09 |
| ME picture type | < 0.0001 |
| *Note.* No effects of the study medication on ratings of drug- or nondrug-related pictures with regard to the picture characteristic craving were discovered. However, there was a significant main effect of picture type, with higher ratings of drug-related pictures as compared to nondrug-related pictures. ME = Main Effect. X = Interaction Term. | |

| Table S2. Descriptive data of cortisol effects on craving related to viewing drug-related and non-drug-related pictures | | | |
| --- | --- | --- | --- |
| Picture type | Low-dose heroin group  (N = 10) | Medium-dose heroin group  (N = 9) | High-dose heroin group  (N = 10) |
| Drug-related pictures | 6.08 (2.5) / 5.42 (2.56) | 4.28 (2.64) / 4.23 (2.76) | 5.1 (2.18) / 5.53 (2.93) |
| Nondrug-related pictures | 2.70 (2.28) / 2.68 (2.78) | 2.08 (2.02) / 1.66 (1.94) | 2.55 (1.34) / 3.74 (2.65) |
| *Note.* Data presented as mean (standard deviation). The first value is indicating the mean and standard deviation of the testing day under placebo, whereas the second value is referring to the testing day under cortisol. | | | |

| Table S3. Co-consumption | | | | | |
| --- | --- | --- | --- | --- | --- |
|  | All (N = 29) | Low-dose heroin group  (N = 10) | Medium-dose heroin group  (N = 9) | High-dose heroin group  (N=10) | *P* |
| Benzodiazepine | 13; 15 | 3; 4 | 2; 3 | 8; 8 | 0.002 |
| Methadone | 9; 11 | 6; 6 | 1; 1 | 2; 4 | 0.006 |
| Amphetamine | 0; 0 | 0; 0 | 0; 0 | 0; 0 | 1 |
| Opiates/Morphine | 29; 29 | 10; 10 | 9; 9 | 10; 10 | 1 |
| THC | 9; 12 | 3; 3 | 3; 4 | 3; 5 | 0.77 |
| Cocaine | 11; 12 | 4; 5 | 3; 2 | 4; 5 | 0.46 |
| *Note.* Positive test results for drug co-consumption for both testing days: the first value indicates the number of positive drug co-consumption for the placebo-testing day, whereas the second value is referring to the medication-testing day.  *P* indicates *p*-values of heroin group effect on drug co-consumption over both testing days. | | | | | |

**Figure S2:** Salivary cortisol concentration over time. The solid line is representing the placebo medication, whereas the dotted line is representing the cortisol medication.
