# Supplementary figures and images for: Effects of cortisol administration on craving in heroin addicts
Source: Transl Psychiatry. 2015 Jul 28;5(7):e610–. doi: 10.1038/tp.2015.101 (PMC5068724; doi:10.1038/tp.2015.101)

Figure S2

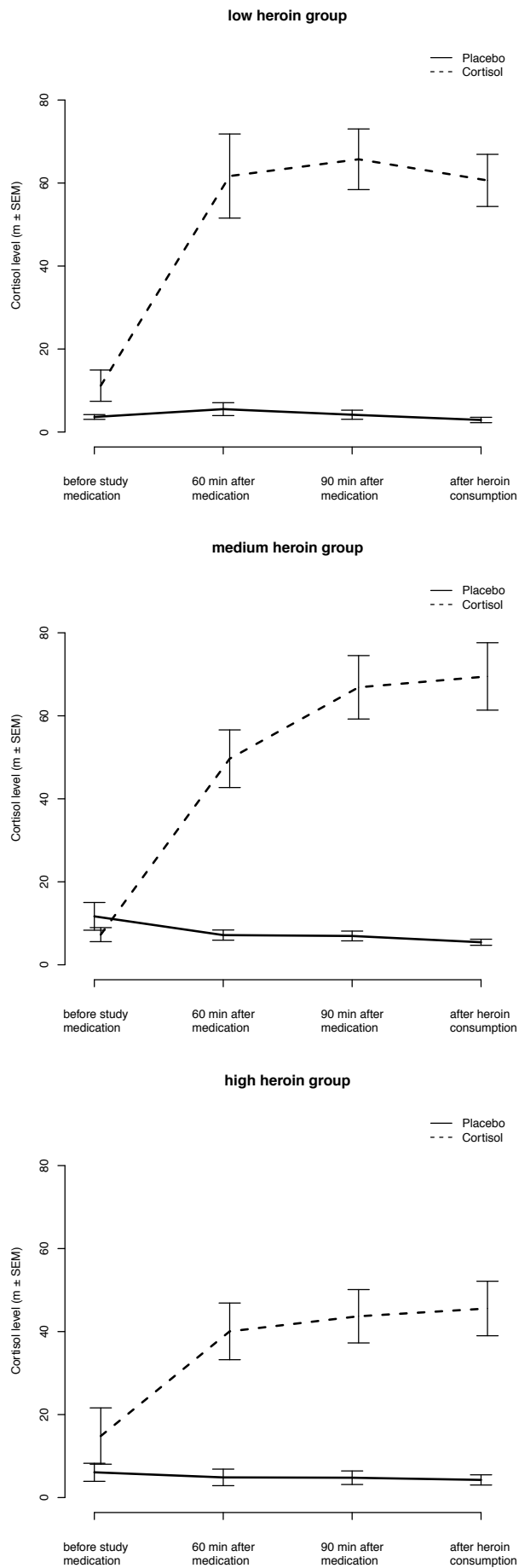

Supplement: Supplementary Figure S2 [file tp2015101x3.pdf]
